# Radiofrequency treatment of cervicogenic headache

**DOI:** 10.4317/medoral.17384

**Published:** 2012-12-10

**Authors:** Maite Bovaira, Miguel Peñarrocha, Maria Peñarrocha, Ana Calvo, Alejandro Jiménez, Rafael March

**Affiliations:** 1 Anesthetist. Supervisor of the Pain Unit of the Levante Recovery and Rehabilitation Center. Valencia. Fellowship in Interventional Pain Practice; 2 Chairman of Oral Surgery, Director of the Master in Oral Surgery and Implantology. University of Valencia Medical and Dental School. Valencia; 3 Associate Professor of Oral Surgery. University of Valencia Medical and Dental School. Valencia; 4 Anesthetist. Staff physician of the Pain Unit of the Levante Recovery and Rehabilitation Center. Valencia; 5 Head of the Department of Anesthesiology. Levante Recovery and Rehabilitation Center. Valencia (Spain)

## Abstract

Objectives: In the clinical management of facial pain, a possible cervical origin must be considered. A clinical exploration is therefore essential. The disorder originates in the intimate connections between the cranial portion of the spinal cord and the trigeminal system. Although solid evidence supporting the use of radiofrequency (RF) treatment is lacking, it remains one of the management options to be taken into account. The present study evaluates the efficacy of RF in application to cervicogenic headache. Study design: We present three cases of severe facial pain arising from different cervical structures. Results: In two cases the pain originated in cervical roots C2 and C3, while in the third patient the trigger point was located at the level of the atlantoaxial joint. Pulsed RF was applied for 4 minutes at the dorsal ganglion of C2 and C3 in the first two cases, and for 8 minutes at intraarticular level in the third patient. The pain gradually subsided during the first month in all cases. The first two patients reported 70% improvement after one month, 60% improvement after 6 months, and 30-50% after one year, versus baseline. The third patient reported complete pain resolution lasting approximately 5 months, after which the pain reappeared with the same intensity as before. Conclusions: Radiofrequency is a satisfactory treatment option, affording adequate analgesia, though the effects are sometimes temporary.

** Key words:**Cervicogenic headache, pulsed radiofrequency, analgesia.

## Introduction

The idea that headache may originate in different neck structures is not new. Barré and Bärtschi-Rochaix described the condition in the first half of the twentieth century (1). The key element distinguishing cervicogenic headache from other migraine syndromes is the concept that the former arises from a structural anomaly in the spinal column or the soft tissues surrounding it. The existence of intimate connections between the cranial portion of the spinal cord and the trigeminal system explains the presence of craniofacial pain of cervical origin (2,3). The different neck structures (facets, disc, roots, muscles and ligaments) are possible sources of pain (3-5). The prevalence of cervicogenic headache varies from 2.5-13.8% (6). Its diagnostic criteria were originally described by Sjaastad in 1998 ( Table 1) (7), and were partially modified by the International Headache Society (IHS) in 2004 ( Table 2) (8). Although solid evidence supporting the use of radiofrequency (RF) applied to the different neck structures is lacking ( Table 3, Table 4), it remains one of the treatment options to be taken into account in pain of this kind (6).

**Table 1:**
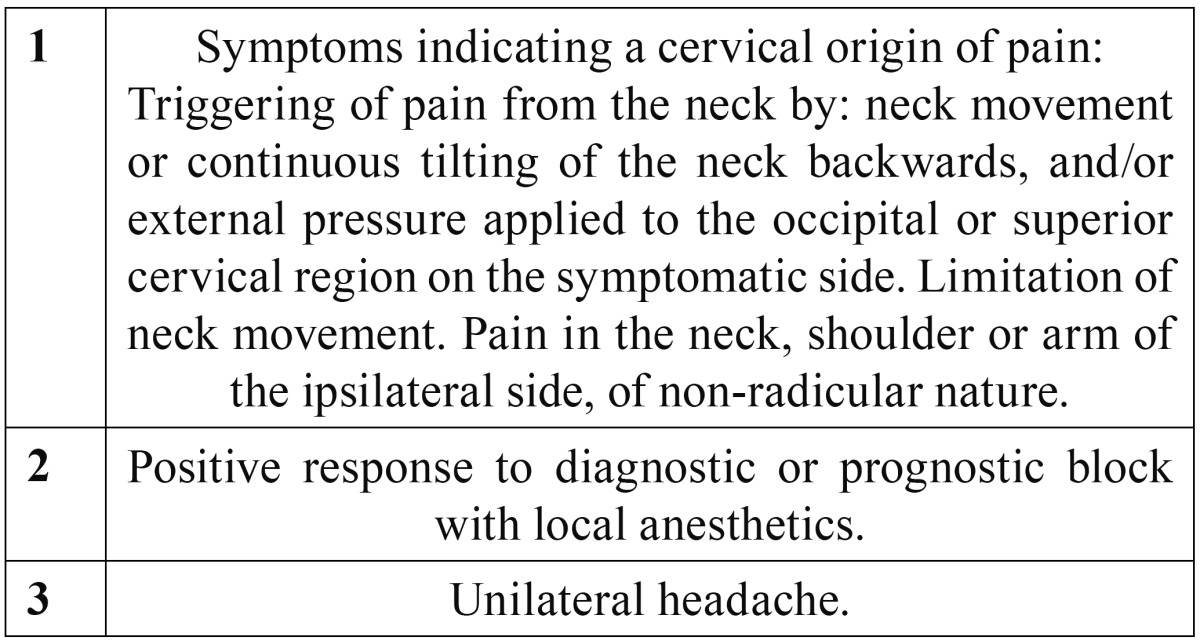
Diagnostic criteria of cervicogenic headache according to Sjaastad.

**Table 2:**
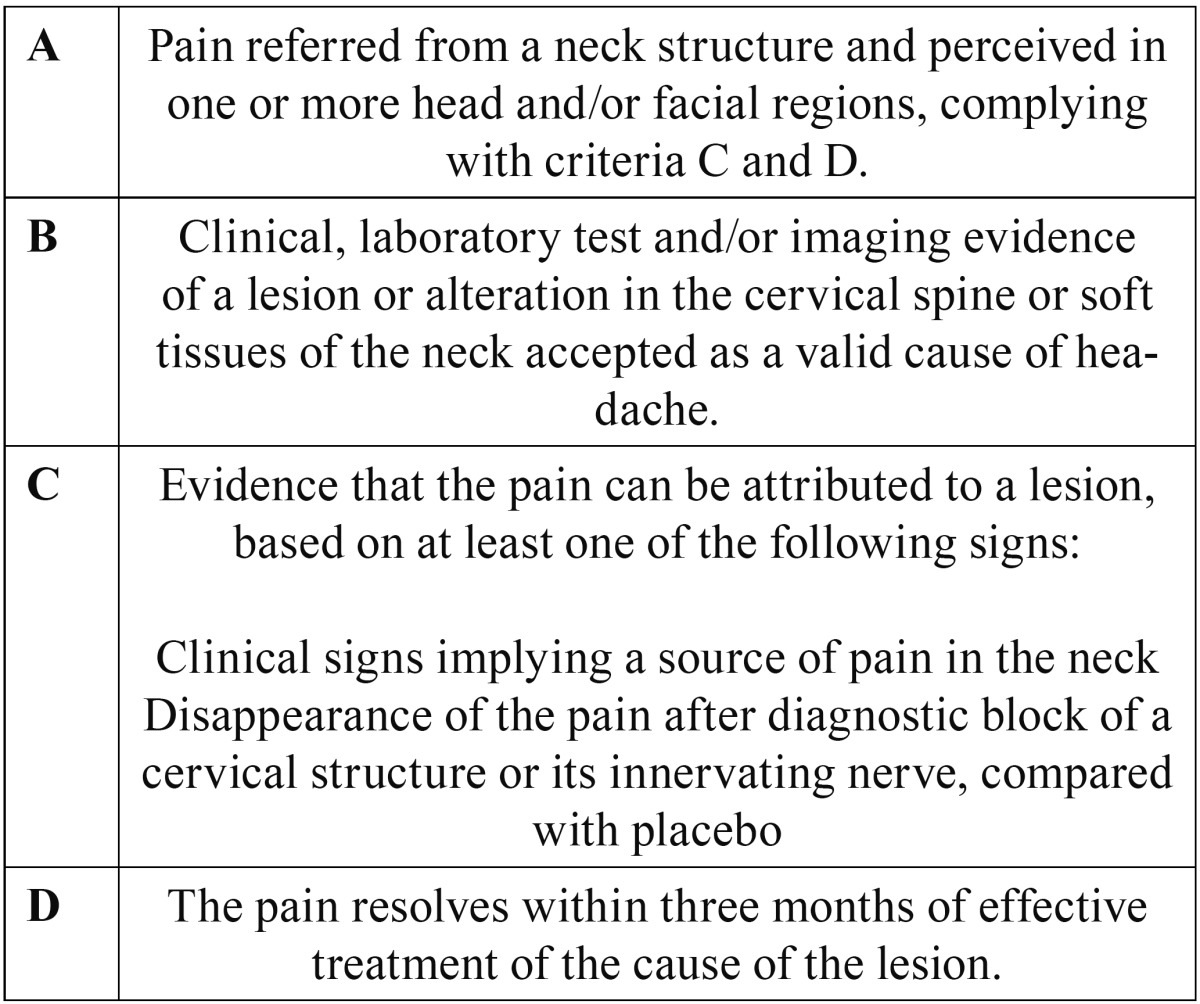
Diagnostic criteria of cervicogenic headache according to the International Headache Society (HIS).

**Table 3:**
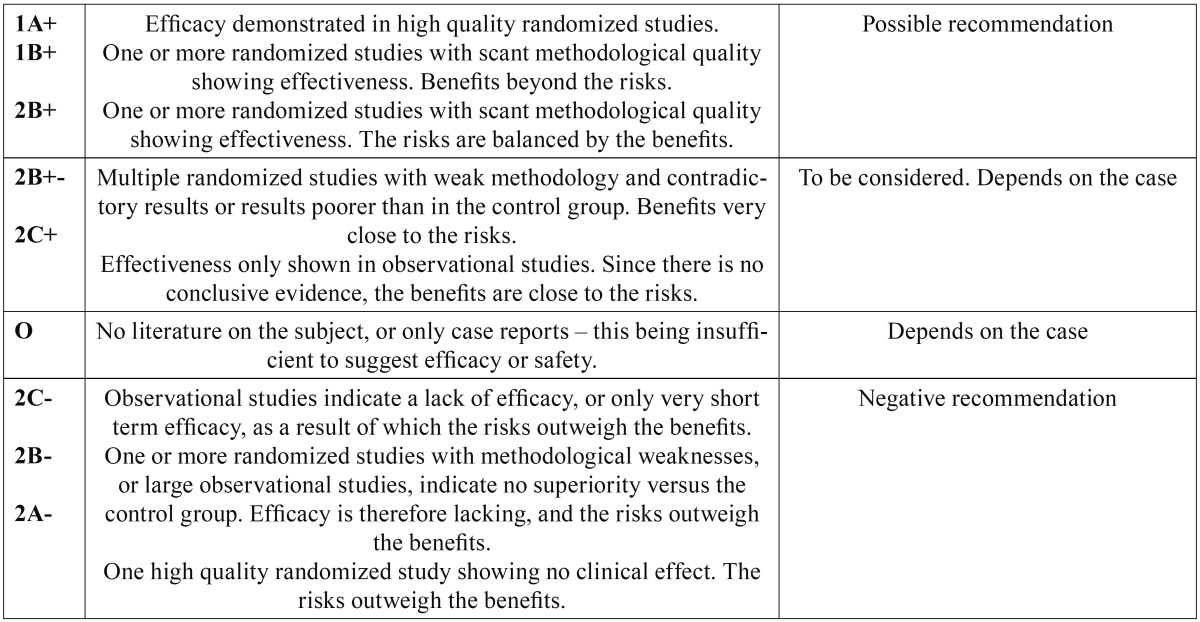
Scale of scientific levels of evidence and recommendations.

**Table 4:**
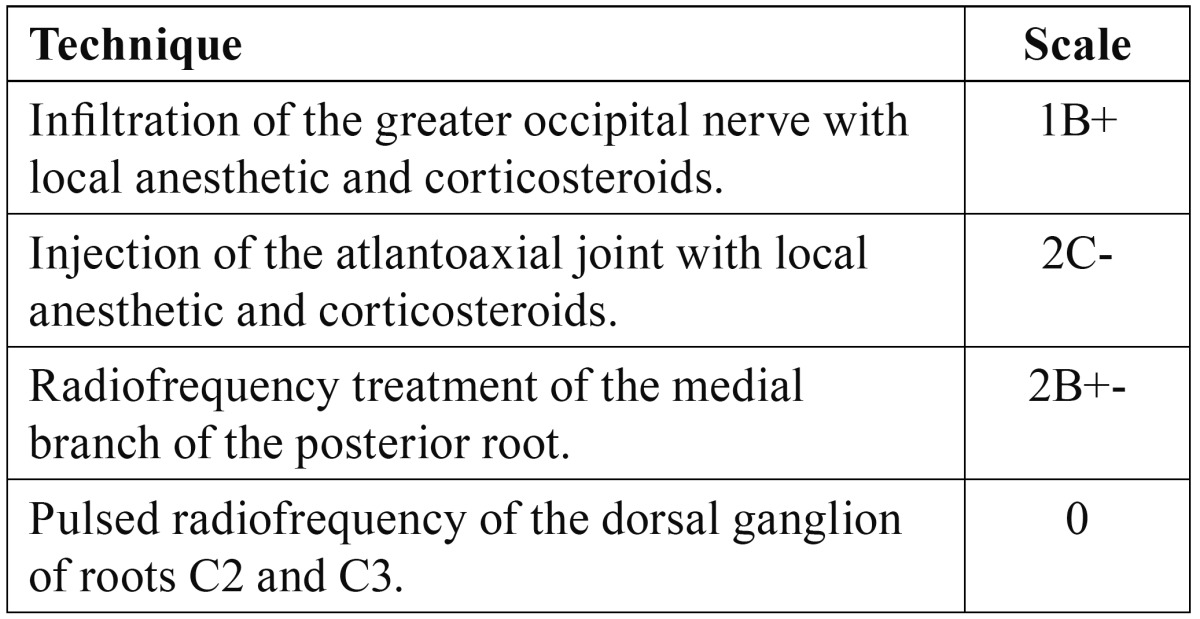
Evidence supporting the treatment options in cervicogenic headache.

The present study describes three cases of severe cervicogenic facial pain subjected to treatment with pulsed RF targeted to the dorsal ganglion of cervical roots C2 and C3 in two cases, and to the atlantoaxial joint in the third patient.

## Material and Methods

Three patients were treated in our Unit due to craniofacial pain of cervical origin, according to the criteria of the International Headache Society ( Table 2).

Case 1: A 64-year-old woman presented with a 17-year history of pain that had worsened in the last year. The pain was constant, with attacks that could last days or weeks, and with a mean intensity of 8/10. It was described as being located in the left periorbital zone, and in the last year additional pain had developed at ipsilateral occipital level, with the limitation of neck movement. The condition showed a clear association to stress, and improved with medication (indomethacin and prednisone). Exploration revealed limitation of left lateral flexion and rotation of the neck, with pain in response to palpation of the left greater and lesser occipital nerves. Treatment consisted of pulsed radiofrequency targeted to the left dorsal ganglion of C2 and C3.

**Figure 1:**
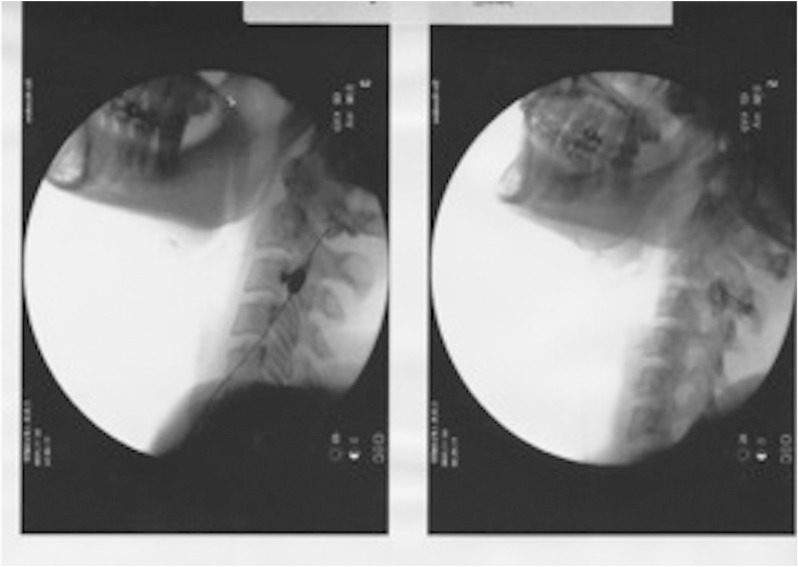
Bilateral pulsed radiofrequency targeted to the dorsal ganglion of roots C2 and C3.

Case 2: A 37-year-old woman presented with bilateral temporomandibular joint dysfunction and bruxomania. She had suffered constant pain during the past year and a half, with occasional episodes of worsening (once a week, and lasting hours), and a mean intensity of 8/10. The pain was described as being located in the preauricular zone, irradiating towards the occipital region and mouth, on both sides of the head, and worsened on speaking and chewing. The patient was taking amitriptyline and ketazolam, and did not tolerate antiinflammatory medication. Physical examination revealed pain in response to bilateral palpation of the temporomandibular joint and greater occipital nerve. The pain worsened with movement of the neck. Cervicogenic headache was diagnosed, and treatment was provided in the form of pulsed radiofrequency targeted bilaterally to the dorsal ganglion of C2 and C3 (Fig. 1).

**Figure 2:**
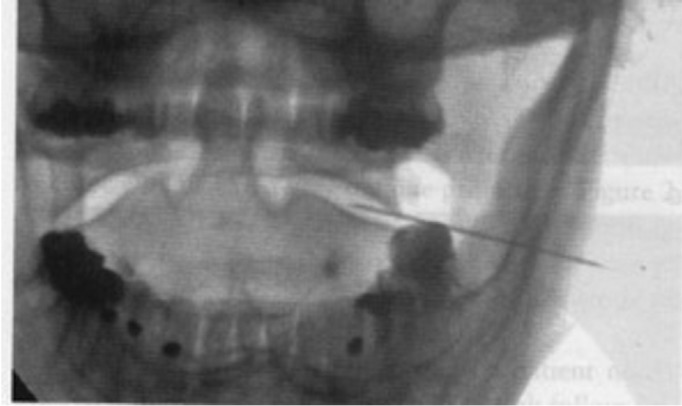
Pulsed radiofrequency of the left atlantoaxial joint.

Case 3: A 27-year-old male with multiple sclerosis diagnosed one year before presented with a 7-year history of pain initially manifesting as regular crises (every 3-4 months) lasting about 7 days, but which had worsened in the last three years, becoming constant with a baseline intensity of 4/10. However, maximum pain scores were reached in the course of 4-5 daily crises. The pain was described as being initially located in the left maxillary and periorbital region, but subsequently displaced towards more posterior cervico-occipital zones, with periorbital irradiation. He had undergone three infiltrations with 4 mg of dexamethasone and 1.5 ml of 3% articaine solution in the sphenopalatine ganglion. The first infiltration afforded total pain relief for 15 days, while the following two infiltrations proved less effective. Treatment was being provided in the form of baclofen (10 mg every 8 hours), indomethacin (25 mg every 8 or 12 hours) and bromazepam (1.5 mg every 24 hours). Physical examination revealed limitation of left rotation and extension of the neck because of the pain, and trigger points were identified in the greater occipital nerve and left atlantoaxial joint region. Treatment consisted of pulsed radiofrequency targeted to the left atlantoaxial joint (Fig. 2).

The patients were followed-up on for a period of one year.

The SPSS version 11.5 statistical package was used to perform a descriptive analysis, while comparisons were made using the Student t-test and analysis of variance (ANOVA).

## Results

**Figure 3:**
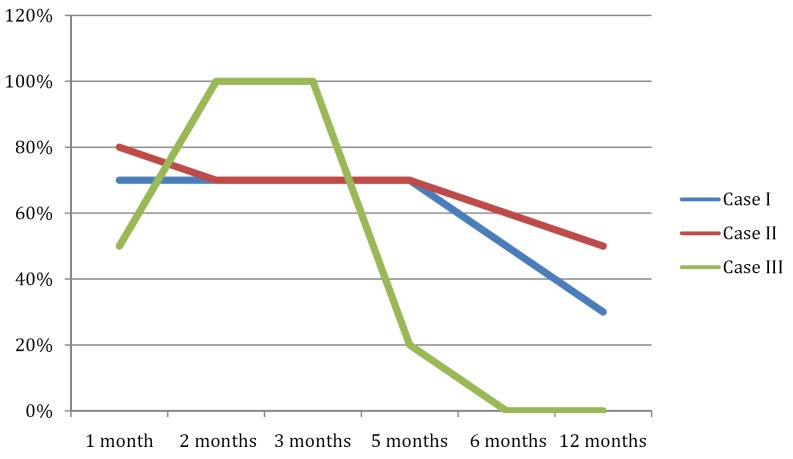
Percentage improvement after radiofrequency treatment.

The pain gradually subsided during the first month in all cases. Patients 1 and 2, subjected to dorsal ganglion RF treatment, reported 70% improvement after one month, 60% improvement after 6 months, and 30-50% after one year, versus baseline. Patient 3, subjected to intraarticular RF therapy, reported complete pain resolution after two months. This benefit lasted approximately 5 months, after which the pain reappeared with the same intensity as before (Fig. 3).

## Discussion

Headache of cervical origin has a considerable incidence of between 2.5-13.8% (5), though in some patients a cervical origin is not considered among the possible causes of the pain. The prevalence is higher among women.

Although the diagnostic criteria define cervicogenic headache as unilateral (7), the condition can also be bilateral (5), as in our 37-year old patient. The pain typically starts in the neck and irradiates towards the frontotemporal zone (patients 2 and 3) or supraorbital region (patients 1 and 3). This situation tends to confuse the diagnosis; as a result, it is very important to perform an exploration of the neck in patients with facial pain, in search of symptoms implicating involvement of the cervical spine, such as limitations in neck movement, and the identification of pain trigger points. In our patients, the clinical manifestations were consistent with cervicogenic headache, i.e., the pain was persistent, non-pulsatile, and was associated to crises of unpredictable duration (hours, days).

Bogduk established that all structures (mainly facet joints, as well as intervertebral discs, muscles and ligaments) innervated by segmental nerves C1, C2 and C3 are possible sources of cervicogenic headache (8). The presence of the trigeminocervical nucleus is essential for explaining the underlying mechanism of action. This nucleus is composed of the pars caudalis of the spinal nucleus of the trigeminal nerve and the gray matter of the three superior segments of the spinal cord, which interact and converge in the second neuron. Thus, neck pain can be perceived in the sensory territory corresponding to the trigeminal nerve (5).

Drug treatment continues to play a fundamental role in these patients. There is no general agreement regarding the treatment of choice for cervicogenic headache, since lack of knowledge of the true mechanism underlying this type of pain obliges us to resort to symptomatic treatments in most cases. There is no solid evidence supporting cervical facet denervation with radiofrequency (RF) or pulsed RF targeted to the dorsal ganglion of the superior cervical roots. At present, the treatment of choice could be regarded as infiltration of the greater occipital nerve (or nerve of Arnold) with local anesthetic and corticosteroids. If this proves ineffective, facet denervation of the highest joints would be the best management choice. A third option in the case of failure of facet denervation would be pulsed RF treatment of the ganglion of the dorsal root of C2 and C3 (6,9,10). There are also other treatments that have opened fields for research, such as intraarticular pulsed RF of the atlantoaxial joint (11,12), pulsed RF of the greater occipital nerve, and even neuromodulation using peripheral electrodes (5).

Our patients had received antiinflammatory treatment, though at the time of the visit these treatments were clearly ineffective, and the patients suffered continuous pain associated to variable episodes of worsening. Only one patient had undergone infiltrations of the sphenopalatine ganglion, with uncertain results. This technique is presently not recommended when a cervical origin of facial pain is suspected. If infiltration is performed, the target should be the greater occipital nerve, and even the atlantoaxial joint. As can be deduced from the above recommendations, RF should be targeted to the medial branch of the posterior root of the cervical spinal nerves, with level of evidence 2B+-, rather than to the dorsal ganglion of roots C2 and C3 – for which the level of evidence is 0 ( Table 3, Table 4). Nevertheless, the latter approach is contemplated in the updated cervicogenic headache treatment algorithm. On the other hand, while intraarticular RF appeared to be a novel and promising treatment option in 2008 (11,12), it has not received much support from posterior studies. Despite the latest proposals referred to invasive treatment in facial pain of cervical origin, we decided to apply techniques that have been described in application to pain of this kind, with acceptable results in the case of pulsed RF of the dorsal ganglion of the posterior root, and currently suboptimal results in the case of intraarticular RF.

In the clinical management of facial pain, a possible cervical origin must be considered. A clinical exploration is therefore essen-tial. Regarding the treatment options, radiofrequency affords adequate analgesia (> 50%), though the effects are sometimes temporary.
